# Predicting clinical trial results based on announcements of interim analyses

**DOI:** 10.1186/1745-6215-15-73

**Published:** 2014-03-07

**Authors:** Kristine R Broglio, David N Stivers, Donald A Berry

**Affiliations:** 1 Berry Consultants, LLC, 4301 Westbank Dr, Suite 140, Bldg B, Austin, TX 78746, USA; 2 Alere, 9975 Summers Ridge Rd, San Diego, CA 92121, USA; 3 University of Texas M.D. Anderson Cancer Center, P.O. Box 310402, Houston, TX 77230, USA

**Keywords:** Predictive probabilities, DSMB, Interim analyses, Operational bias, Prior distribution, Bevacizumab, Colon cancer

## Abstract

**Background:**

Announcements of interim analyses of a clinical trial convey information about the results beyond the trial’s Data Safety Monitoring Board (DSMB). The amount of information conveyed may be minimal, but the fact that none of the trial’s stopping boundaries has been crossed implies that the experimental therapy is neither extremely effective nor hopeless. Predicting success of the ongoing trial is of interest to the trial’s sponsor, the medical community, pharmaceutical companies, and investors. We determine the probability of trial success by quantifying only the publicly available information from interim analyses of an ongoing trial. We illustrate our method in the context of the National Surgical Adjuvant Breast and Bowel (NSABP) trial, C-08.

**Methods:**

We simulated trials based on the specifics of the NSABP C-08 protocol that were publicly available. We quantified the uncertainty around the treatment effect using prior weights for the various possibilities in light of other colon cancer studies and other studies of the investigational agent, bevacizumab. We considered alternative prior distributions.

**Results:**

Subsequent to the trial’s third interim analysis, our predictive probabilities were: that the trial would eventually be successful, 48.0%; would stop for futility, 7.4%; and would continue to completion without statistical significance, 44.5%. The actual trial continued to completion without statistical significance.

**Conclusions:**

Announcements of interim analyses provide information outside the DSMB’s sphere of confidentiality. This information is potentially helpful to clinical trial prognosticators. ‘Information leakage’ from standard interim analyses such as in NSABP C-08 is conventionally viewed as acceptable even though it may be quite revealing. Whether leakage from more aggressive types of adaptations is acceptable should be assessed at the design stage.

## Background

Interim analyses of clinical trials convey information beyond the sphere of confidentiality of a trial’s Data and Safety Monitoring Board (DSMB). A press release from the sponsor may announce, ‘Good news! The DSMB recommends that the trial continue,’ or the opposite reaction: ‘Shares of Oncothyreon dove over 40% on Tuesday after the [announcement] that a phase III clinical trial for Stimuvax [would] continue for several more months’ [1]. Such public announcements indicate that the results have not crossed a stopping boundary. An implication is that the experimental therapy is neither extremely effective nor hopeless nor obviously unsafe. In the case of Stimuvax, Wall Street had convinced itself that the product was so good that crossing an early efficacy boundary was a foregone conclusion [2].

Trial sponsors are obviously interested in the results of interim analyses. Other interested parties include trial investigators, patients, venture capitalists, stockbrokers, and the sponsor’s competitors. When processing announcements of DSMB recommendations, and if left to their own devices, people process information badly, usually overestimating or underestimating. In the words of Daniel Kahneman, ‘intuitive impressions of the diagnosticity of evidence are often exaggerated’ [3]. We illustrate a methodology for incorporating only the publicly available results of interim decisions in order to find the probability the trial will be successful. We assumed here that the interim analysis rules from the trial’s protocol are available to the assessor.

Finding the probability of trial success based on interim information requires the probability of success separate from, or prior to, that information. The probability of trial success based on the prior and currently available information, and not assuming any particular hypothesis about treatment effects is called ‘predictive power,’ and contrasts with both traditional power and conditional power. Traditional power, assessed at the design stage, assumes a particular ‘clinically significant’ treatment effect [4]. Conditional power, assessed at an interim point in the trial, conditions on the current data, and assumes a particular point estimate (usually the maximum likelihood estimate) for the treatment effect. Both traditional power and conditional power may be considered across a variety of scenarios, but the assumed treatment effect in each scenario is fixed. By contrast, predictive power is a weighted average of power over the current ‘posterior’ distribution of the unknown treatment effects [5].

The distribution of treatment effects prior to observing any information from the trial; that is, the prior distribution, depends on phase 2 results; evidence from trials involving similar treatments, such as drugs in the same class; and evidence from trials with the same treatment but different diseases. It also depends on the person making the assessment, which gives the Bayesian approach an explicitly subjective flavor. One person may be pessimistic about successfully developing a treatment for a particular disease, whereas another may be optimistic. The hypothesized effect used in powering a clinical trial tends to be optimistic; for example, it may be based on the results of phase 2 trials. Such an estimate is biased and subject to regression to the mean [6,7]. Evidence for this statement is that phase 3 clinical trials have at least 80% power, and yet no therapeutic area achieves a success rate even approaching this level [8].

An honestly assessed prior distribution allocates some probability to treatment effects that are less optimistic than the one used in powering the trial. It might also assign some probability to more optimistic treatment effects. Perhaps the prior distribution is symmetric about its mean, and the mean is the effect at which the trial is powered. The excess in power above the mean does not begin to compensate for the loss in power below the mean. Therefore, properly incorporating uncertainty, the predictive calculations give a probability of success that is less than the calculated power. An empirically based rule of thumb is that trials built to have 80% power have a success probability of 60%. Moreover, the mean of the prior distribution is usually too optimistic. Discounting for excessive optimism decreases predictive power still more, to even less than 50%.

We illustrate our methodology using the National Surgical Adjuvant Breast and Bowel Project trial C-08 (NSABP C-08) [9]. This trial evaluated the benefit of adding bevacizumab (Avastin®; Genentech/Roche) to modified infusional fluorouracil, leucovorin, and oxaliplatin (mFOLFOX6) in treating colon cancer.

## Methods

### NSABP C-08 study design

NSABP C-08 randomized patients with stage II or III colon cancer equally to mFOLFOX6 (control group) versus bevacizumab plus mFOLFOX6 (experimental group). The primary endpoint was disease-free survival (DFS) measured from randomization to the first recurrence, second primary cancer, or death from any cause. Based on previous studies, the control event rate was expected to be highest in the first 3 years. In total, 2,632 patients were planned to give 90% power to detect an anticipated 25% reduction in the hazard of recurrence. The primary analysis would be a one-sided log-rank test.

The trial’s target monthly accrual rate was expected to increase from 63 to 105 patients over the first 2 years, completing accrual within 30 months. The protocol-defined final analysis was to occur at 592 DFS events. The first interim analysis was planned after 148 DFS events (25% information), approximately 2 years after trial initiation. Subsequent analyses were planned every 6 months. Neither the number of analyses nor the significance level of the final analysis were predetermined, but would depend on alpha spending [10]. However, the investigators anticipated six interim analyses, which is what we assumed in our simulations. The stopping rule used asymmetric boundaries for superiority and futility indexed by one-sided *P*-values (Table 1). Maximal trial duration was expected to be 5 years, including 2.5 years of follow-up after the final patient was accrued.

**Table 1:** **National Surgical Adjuvant Breast and Bowel Project trial C-08 (NSABP C-08) interim monitoring bounds in terms of one-sided *P*-values**

**Interim analysis**	**Estimated time, years**	**Stop for futility if right-sided *P*-value <**	**Stop for superiority if left-sided *P*-value <**	**Number of events**	**Estimated accrual at interim**
1	2.0	0.05	0.00025	148	2,006
2	2.5	0.25	0.0005	~220	2,632
3	3.0	0.5	0.001	~312	2,632
4	3.5	0.5	0.001	~398	2,632
5	4.0	0.5	0.001	~473	2,632
6	4.5	0.5	0.001	~538	2,632
Final	5.0	N/A	~0.0246	592	2,632

### Statistical simulations

We simulated trial results based on the publicly available specifics of the NSABP C-08 design and the results of previous interim analyses. We assumed particular treatment effects (see below) to generate virtual patients based on the assumed accrual rates, and followed their DFS events over time. These virtual patients experienced DFS events over time, depending on the assumed effect of their assigned treatment. Each simulated trial stopped or not at interim analyses, according to the boundary defined in Table 1. If it did not stop early then we compared the log-rank *P*-value to the final one-sided significance level of 0.0246, as shown in Table 1. When a simulated trial stopped at an interim analysis we recorded that fact and the result. For each simulation, we recorded whether, when, and why the trial stopped, and the eventual statistical conclusion. We iterated this process 70,000 times for each assumption about treatment effects. The proportion of simulated trials with outcomes of interest (such as that the trial continues at the third interim analysis, but stops for superiority of the experimental arm at the fourth interim analysis) are the estimated probabilities of those outcomes.

We followed the assumption in the trial’s protocol when generating the time that each virtual patient would experience a DFS event. Specifically, we assumed a piecewise exponential distribution with control hazard rate 0.089 for the first 3 years, reducing to 0.039 in subsequent years. When generating patients in the bevacizumab group, we assumed a constant hazard ratio versus control.

**Figure 1:**
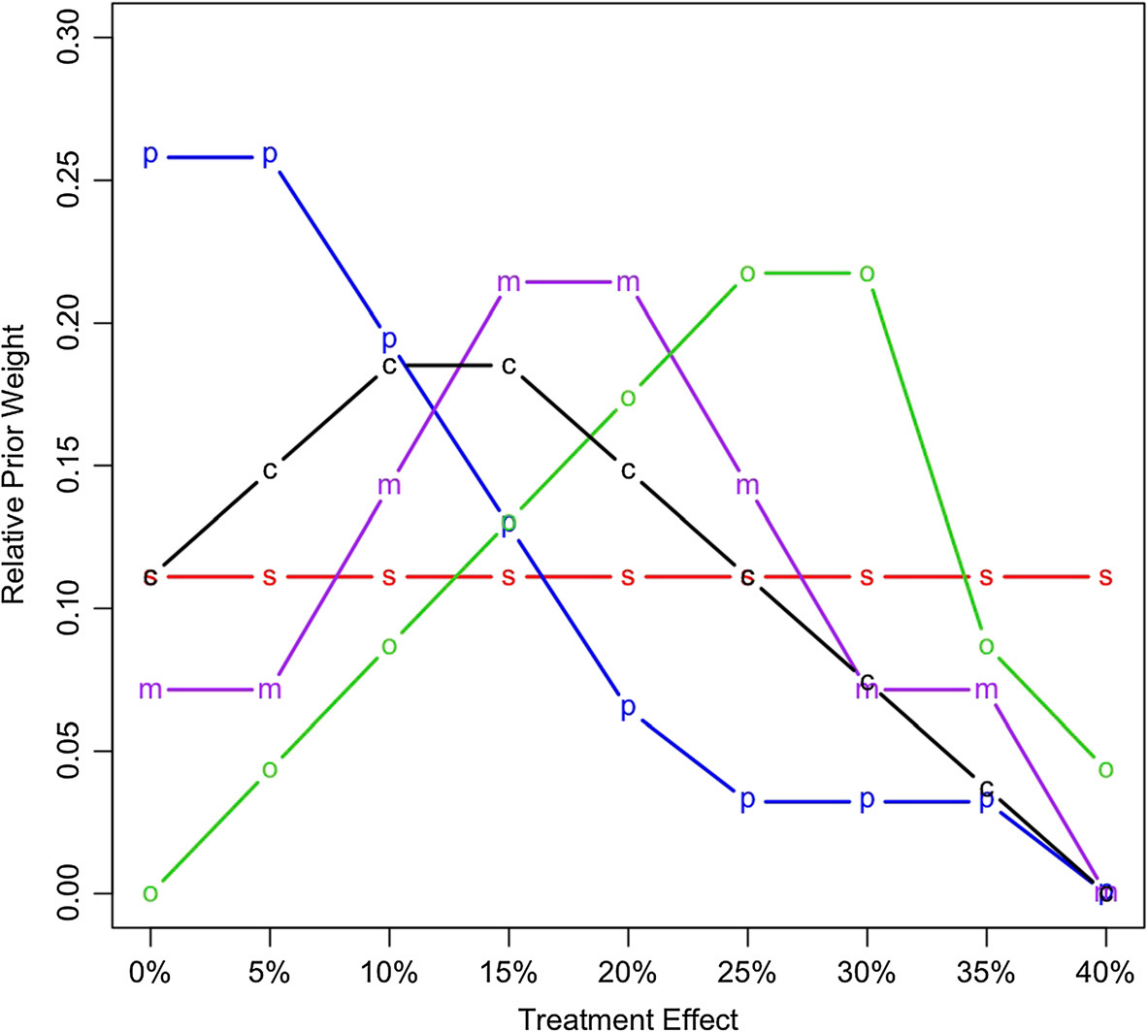
**Prior distributions of the treatment benefits given in terms of relative strengths of belief in hazard reduction due to bevacizumab.** Abbreviations s, simple; p, pessimistic; o, optimistic; m, moderate; and c, custom.

The treatment effect was the reduction in hazard due to bevacizumab. Any value from negative infinity to 100% was possible, but the practical range was 0 to 40%. We assigned (prior) weights to values in this range, in increments of 5%. Figure 1 gives example relative weights.

The ‘custom’ prior distribution in Figure 1 was that assessed (by author DAB) during the NSABP C-08 trial. This prior was not based on any knowledge of trial results, but considered publicly available information from other colon cancer trials and from other trials of bevacizumab [9,11-15]. The available information was consistent with reductions of 10% and 15%, with little suggestion that the reduction could be 30% or greater.

The other prior distributions shown in Figure 1 were used to address the sensitivity of our conclusions to the prior assessments. ‘Simple’ gave equal weight to each possible reduction; ’pessimistic’ concentrated weight on little or no reduction; ‘moderate’ focused on 15% and 20% reductions; and ‘optimistic’ gave most weight to substantial reductions.

## Results

### Prior predicted power

**Figure 2:**
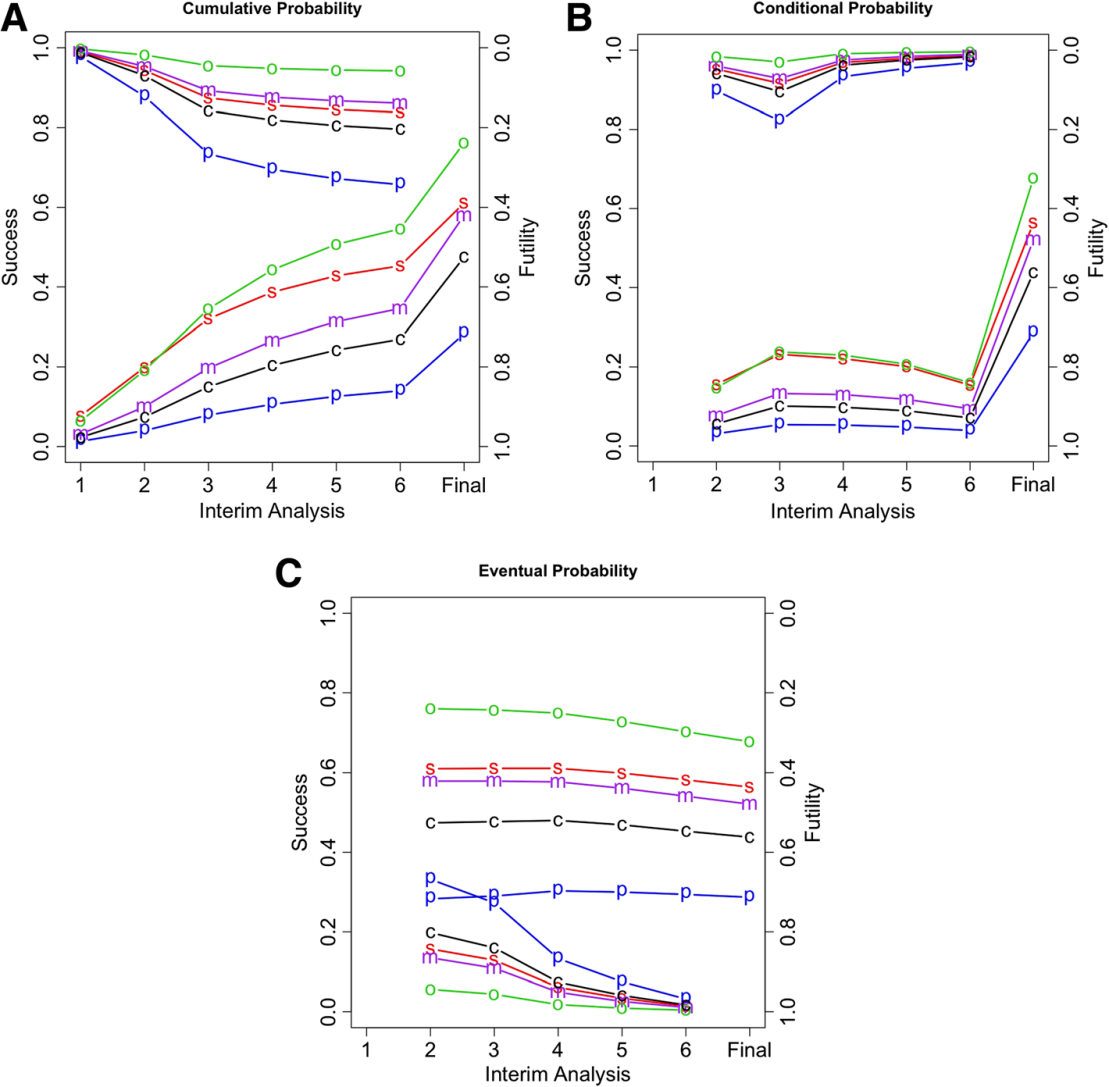
**The symbols use in all panels refer to the prior weights indicated in Figure**1**,’ Abbreviations s, simple; p, pessimistic; o, optimistic; m, moderate; and c, custom.** The top five lines and right *y*-axis show the futility results. The bottom five lines and the left *y*-axis show the success results. **(A)** Predictive probability of success or futility at or before the indicated interim analysis. **(B)** Probability of success or futility at the indicated interim analysis conditionally on ‘Continue’ at the previous interim analysis. **(C)** Predictive probability of eventual success or futility assuming ‘Continue’ at the previous interim analysis.

Figure 2(A) shows the cumulative predictive probability of success (bottom five lines) and the cumulative predictive probability of futility (top five lines). The steep increase in the cumulative probability of success at the final analysis reflects the larger *P*-value boundary used at the end of the trial compared with those used for interim analyses. Based on the custom prior distribution, the overall probability of statistical success was 47.5%, much less than the trial’s advertised power of 90%. The predictive probability of success is substantially lower than the power because it incorporates uncertainty in the hazard reduction, and gives weight to hazard reductions of less than 25% (at which power was evaluated). The cumulative probability of success by the final analysis based on the ‘optimistic’ prior was 76%, which was still less than the trial’s planned power. This is despite the fact that this prior distribution was centered close to the 25% reduction assumed in the protocol. The decrement in the probability of success is due to the uncertainty that is explicitly considered in the prior distribution.

The increases in the cumulative probability of futility from the first and third interim analyses reflect the less stringent futility stopping bounds subsequent to the second interim analysis; futility is a possibility only at interim analyses. Based on the custom prior, the overall probability of futility was 20.4%. This ranged from 5.8% with the optimistic prior, to 34.3% with the pessimistic prior. The cumulative custom probability the trial would remain undecided at the final analysis was 32.1%. The cumulative probability of ‘undecided’ ranged from 18% for the optimistic prior, to 37.4% for the pessimistic prior.

Figure 2(B) shows the predictive probability of success and the predictive probability of futility, conditional on the previous interim analysis decision being to continue. Figure 2(C) shows the predictive probabilities of eventual success and the predictive probabilities of eventual futility. Figure 2(C) indicates that the probability of eventual success in the trial, given that it continues, decreases only slightly over time. In this sense, announcing the DSMB’s recommendation to continue is not very informative.

### Actual trial conduct

**Figure 3:**
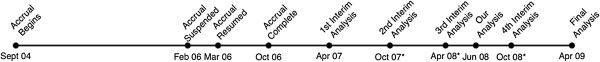
**Timeline of actual National Surgical Adjuvant Breast and Bowel Project trial C-08 (NSABP C-08) trial.** Asterisk indicates approximate timing.

Figure 3 shows the timeline of NSABP C-08 and our predictions. The trial began in September 2004 [9]. In February 2006, accrual was temporarily halted to allow the DSMB to review adverse event data. Accrual resumed in May 2006. Accrual was faster than expected, and was completed in October 2006 with 2,710 patients enrolled [9]. The first interim analysis was conducted in April 2007.

Our analysis of the predictive probability of the trial’s success was conducted in June 2008, after the third interim analysis. We made our software and ‘custom’ prior distribution public on June 12, 2008 via a webinar sponsored by UBS Bank. Our predictive probabilities for the fourth interim analysis were 9.8% for success and 3.8% for futility. At that time, our predictive probabilities of eventual success, stopping early for futility, and continuing to completion without statistical significance were 48.0%, 7.4%, and 44.5%, respectively.

**Figure 4:**
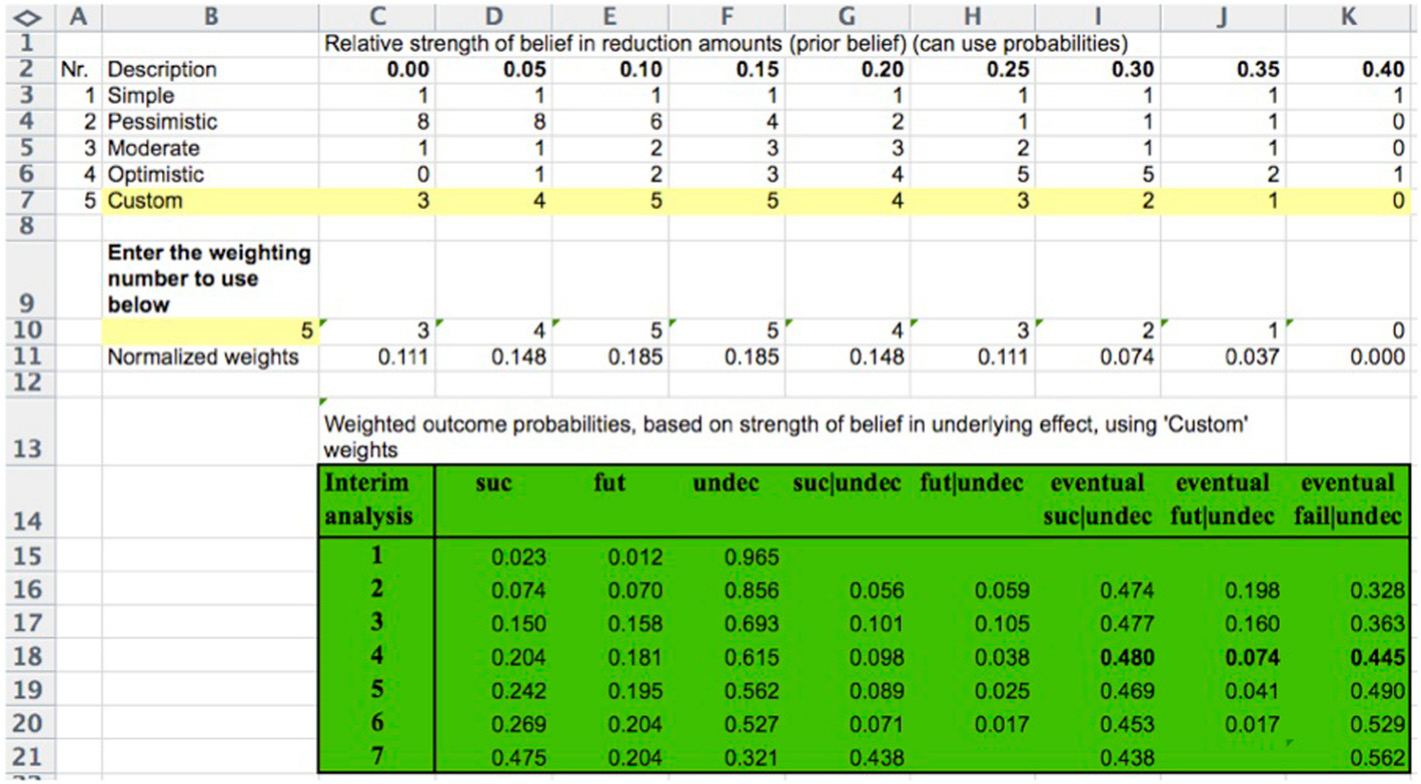
**Screenshot of Excel spreadsheet demonstrating the calculations described in the text.** Entry ‘5’ at cell 10B refers to the entry in cell 7A, and sets the prior probabilities to those labeled ‘Custom’ in row 7. These were the probabilities of the author (DAB) during the early conduct of National Surgical Adjuvant Breast and Bowel Project trial C-08 (NSABP C-08), and were based on the publicly available information about bevacizumab and its potential efficacy in treating adjuvant colon cancer, as described in the text. ‘Interim analysis 7’ is actually the final analysis. The emboldened type in row 18 indicates the probabilities of eventual success, futility, and failure after the fourth interim analysis. This spreadsheet is available at http://www.berryconsultants.com/wp-content/uploads/2014/02/Unknown-Hazard-Reduction.xls. It allows users to input their own probabilities, and to experiment with other prior probabilities to see how they affect conclusions. The column headings are ‘suc’ (the probability of success at the current row’s interim analysis); ‘fut’ (the probability of futility at the current row’s interim analysis); ‘undec’ (the probability of making no decision at the current row’s interim analysis and continuing the study); ‘suc|undec (the probability the trial will be declared successful at that row’s interim analysis, given no decision at any previous analysis); ‘fut|undec (the probability the trial will be declared futile at that row’s interim analysis, given no decision at any previous interim analysis); ‘eventual suc|undec (the probability the trial will be declared successful at the current, future, or final analysis, given no decision at any previous analysis); ‘eventual fut|undec (the probability the trial will be declared futile at the current, future, or final analysis, given no decision at any previous analysis); and ‘eventual fail|undec (the probability the trial will fail to show statistical significance given no decision at any previous analysis).

Our analysis depended on the assumed prior probabilities, the roots of which are subjective [16]. An Excel spreadsheet that carries out the calculations described in this article is available at http://www.berryconsultants.com/wp-content/uploads/2014/02/Unknown-Hazard-Reduction.xls (Figure 4). It allows the user to input a prior distribution by entering a weight for each of the indicated hazard reductions. The sum of the weights is arbitrary; only their relative sizes matter. The weights convey confidence in the corresponding hazard reductions, and should reflect the available evidence. For example, entering ‘10’ for the 25% reduction and ‘5’ for the 0% reduction means that the prior belief in the former is twice as strong as for the latter. Once the prior distribution is entered, the probabilities of success and futility and other conditional measures are calculated and displayed. Varying the prior weights shows how the probabilities of the various outcomes depend on these weights. For example, entering zero weight in all but a 25% reduction gives the overall power of the trial evaluated at this ‘clinically important difference’, which is 92%.

Owing to more rapid accrual than expected, the actual NSABP C-08 had only four rather than the planned six interim analyses [9]. The trial continued to completion, and the final analysis, conducted in April 2009, showed that the bevacizumab arm reduced the hazard for DFS events by 11%. This was not statistically significantly different from control (hazard ratio 0.89, 95% confidence interval 0.76 to 1.04) *P*-value = 0.15) [9]. This result was consistent with our custom prior, which gave the most weight to a 10 to 15% reduction in hazard.

## Discussion

Predicting whether an ongoing clinical trial will be successful is of interest to the trial sponsor, the scientific community, other pharmaceutical companies, and investors. When the NSABP C-08 trial was initiated, the owner of bevacizumab was Genentech. As the trial proceeded, Hoffman-LaRoche was in negotiations to purchase the 50% portion of Genentech stock that it did not already own. Hoffman-LaRoche became aware of our software as shown in Figure 4, and they contracted with us to update the software using the actual accrual from the trial (data not shown, and not publicly available at the time) rather than using accrual projected from the protocol.

Hoffman-LaRoche had access to our software after the fourth interim analysis. Using their prior probabilities, they concluded a 55% probability of trial success and used that prediction to set the price they offered for the Genentech stock [17]. Genentech determined the probability of trial success to be 61% [17]. We do not know the basis of this assessment, but it is close to that of the moderate prior defined in Figure 1, and it too is substantially less than the trial’s power of 90% claimed in the protocol (which assumed a 25% reduction in hazard).

We used the NSABP C08 trial as an example to illustrate a general approach. Not stopping at an interim analysis provides some information about the trial’s primary endpoint and its eventual success. The amount of information depends on the trial’s stopping boundaries, and is usually small. We demonstrated how the information gained from the announcement of an interim analysis that the trial is continuing can be used to make and update predictions about the final trial conclusion.

NSABP C08 had more planned and actual interim analyses than is typical in phase III trials. A greater number of interim analyses provides more opportunity for updating, but not necessarily more overall interim information. The number of opportunities for updating may not be the same in other phase III trials, but the approach and the methodology is the same. Moreover, as regards efficacy, and assuming that the total amount of type I error allocated to interim analyses is similar, the total amount of information gleaned from interim analyses is also similar. The difference for another trial is that the information may be revealed at a different rate over time.

Trial designs vary more in their approach toward futility stopping than they do in their approach toward efficacy stopping. The information from an interim analysis for futility is positive in the sense that not stopping bodes better for trial success, although the amount of information may range from minimal to substantial. When stopping for either efficacy or futility are both possibilities at the same analysis epoch, as in the case of NSABP C08, then the information that the trial is continuing could be either positive or negative in relation to ultimate success.

If a trial has no interim analyses, then no updating is possible. However, even then it is possible to calculate the predictive probability of trial success using prior information about treatment effect.

As our example NSABP C08 suggests, the assessment of prior probabilities of the effect of experimental therapy is crucial, and prior information is usually more important than information that accrues from interim analyses. Assessing prior probabilities is inherently subjective, as we demonstrated by providing a variety of prior distributions. In practice, such variety will usually exist, and there may be even a wider range. In our experience, the worst assessors of prior probabilities tend to be those closest to the development of the experimental therapy; sometimes they have been ‘entrapped’ [6]. In our ‘custom’ prior we considered the performance of bevacizumab in other trials of colorectal cancer, trials of bevacizumab in other cancers, trials in cancers involving similar agents, and the sensitivity of colorectal cancer to therapies more generally. This last point is crucial. It plays the dominant role in predictions involving diseases such as pancreatic cancer, glioblastoma multiforme, stroke, sepsis, and Alzheimer’s, where the overwhelming proportion of phase III trials have failed.

Having software that quantifies the information contained in announcements of interim analyses raises concerns about ethics and the need to preserve clinical trial integrity. Our approach does not provide more information, but rather indicates how to interpret the available information. In our experience, interpretations of announcements of interim analyses are frequently wrong, and are usually overinterpretations, sometimes in the wrong direction. This applies to the trials’ investigators and other experts in the field as well as to venture capitalists and Wall Street bankers. Having software that provides a more accurate and usually more tempered interpretation may have a beneficial and moderating effect.

Although our main focus is on outside observers, software that accurately assesses the extent of ‘leakage of information’ in a clinical trial’s design may be an aid to clinical trial designers and trial review bodies. Our software could help them assess the extent and impact of information leakage on the trial. No leakage is good, but some leakage is necessary. The benefits of interim analyses to patients and to society generally usually greatly outweigh the negative aspects of information leakage, but assessing the extent of leakage may lead to a modification of the design that lessens its potential impact on the trial’s conduct.

In a related vein, modern trialists are introducing adaptations that are more complicated than group-sequential methods [18,19]. If these adaptations become public during the trial, and if the trial design is similarly known, then the observed changes in the trial may convey information about the relative performance of the treatment arms [20-22]. Trialists should assess the impact of such knowledge outside the sphere of confidentiality of the trial’s DSMB, and may want to take steps to keep adaptations confidential or perhaps modify the design.

## Conclusions

Announcements of interim analyses for a group-sequential trial design such as NSABP C-08 reveal that the interim data lie within a particular range. This information is potentially helpful to clinical trial prognosticators and to clinical trial designers. Interim analyses such as those in NSABP C-08 are typically viewed as acceptable with regard to the amount of information conveyed [18,22]. Not all prognostication is done well, but any prognostication has the potential to influence the course of the trial. Our formal procedure based on Bayesian updating uses information that becomes available over time, in order to assess accurately the probability that a clinical trial will be positive.

## Competing interests

The calculations reported here were carried out during the trial based on publicly available information. When our analyses were made public during the trial, Hoffman-LaRoche funded a subsequent analysis by Berry Consultants, based on the actual trial accrual rates over time.

## Author contributions

KRB contributed to data interpretation and drafted the manuscript. DS carried out the simulation study, programmed the Excel spreadsheet, and contributed to writing the manuscript. DAB conceived and designed the study, and contributed to writing the manuscript. All authors read and approved the final manuscript.
